# MOBILE APP-BASED SURVEY DEPLOYMENT PATTERNS AND LONGITUDINAL ENGAGEMENT: A RANDOMIZED CONTROLLED TRIAL

**DOI:** 10.1093/geroni/igae098.2903

**Published:** 2024-12-31

**Authors:** Yuankai Zhang, Jian Rong, Xuzhi Wang, Eric Schramm, Chathurangi Pathiravasan, Ludovic Trinquart, Chunyu Liu, Joanne Murabito

**Affiliations:** Boston University School of Public Health, Boston, Massachusetts, United States; Boston University Chobanian & Avedisian School of Medicine, Boston, Massachusetts, United States; Boston University School of Public Health, Boston, Massachusetts, United States; Care Evolution, Ann Arbor, Michigan, United States; Boston University School of Public Health, Boston, Massachusetts, United States; Tufts University, Medford, Massachusetts, United States; Boston University School of Public Health, Boston, Massachusetts, United States; Chobanian and Avedisian School of Medicine, Boston University, Boston, Massachusetts, United States

## Abstract

Smartphone apps are increasingly used to collect digital survey data. Limited evidence is available on effective strategies to improve response rates especially among older adults. We conducted a randomized controlled trial (RCT, NCT04752657) embedded in the electronic Framingham Heart Study (eFHS) to test the effect of administering half of smartphone surveys every 2 weeks (experimental group) compared to administering all surveys every 4 weeks (control group) on participant response rate over 32 weeks. In all, 492 participants (mean age 73 years, 58% women, 16% non-White) were randomized. The primary outcome was the proportion of surveys returned per participant assessed longitudinally across four periods (baseline to week 8, week 8 to 16, week 16 to 24, week 24 to 32). We used mixed-effect regression models with random intercept to compare the mean proportions between groups across the 4 periods, adjusting for age (≤75 versus >75 years) and phone type (Android versus iPhone) that were used to stratify randomization. We observed significant effect modification by time (P-value=0.003), indicating that the difference in response rates between the two groups increased over time. The response rates decreased over time in both groups, but less so in the experimental group with a 7% point difference in the last period. The mean outcome decreased from 85% to 66% in the experimental group, and from 85% to 59% in the control group across the four time periods. This RCT demonstrated that the survey deployment patterns impacted the response rates over 32 weeks among older adults.

